# Diverticular disease: obesity and its impact in the development and treatment of early onset diverticulitis

**DOI:** 10.51894/001c.144455

**Published:** 2025-09-30

**Authors:** Keyla Galloza Acevedo

**Affiliations:** 1 Department of Internal Medicine Detroit Medical Center-Sinai Grace Hospital

## 78

### INTRODUCTION

Diverticulitis, a common cause of hospitalization in the United States, has traditionally been associated with older adults. However, recent trends indicate a rising incidence among younger populations, particularly those with obesity. This case report explores the role of obesity as a risk factor in the development and progression of diverticulitis in younger patients and highlights the challenges in managing complicated cases.

### CASE PRESENTATION

We present a 36-year-old obese African American female with a history of recurrent diverticulitis and endometrial cancer. The patient presented with severe left lower quadrant abdominal pain, nausea, and vomiting. The patient had initially been seen at another hospital prior to admission, after presenting with similar complaints. Imaging revealed chronic active diverticulitis of the descending-sigmoid junction, complicated by diverticular abscess. Patient was discharged home with antibiotics, however, was non-complaint with treatment. CT imaging in current admission revealed complicated diverticulitis with abscess formation and contained perforation. Despite initial management with IV antibiotics, the patient experienced persistent symptoms and required percutaneous drain placement and eventual surgical intervention due to complications, including a colo-atmospheric fistula and necrotizing abdominal soft tissue infection. The patient’s treatment involved a multidisciplinary approach, including consultations with colorectal surgery, gastroenterology, interventional radiology, and infectious disease specialists. Management included IV antibiotics, percutaneous drain placement and surgical debridement. Surgical intervention was necessary to manage the complications, and the patient eventually underwent a successful sigmoid colectomy with end-to-end anastomosis.

### DISCUSSION

Obesity is increasingly recognized as a significant risk factor for the development of diverticulosis and diverticulitis in younger populations. The pathophysiological mechanisms may involve inflammatory cytokines and alterations in gut microbiota. This case underscores the importance of timely diagnosis, strict adherence to antibiotic regimens, and a multidisciplinary approach in managing complicated diverticulitis. Further research is needed to elucidate the mechanisms by which obesity contributes to diverticulosis and to develop targeted prevention and management strategies.

### CONCLUSION

This case report highlights the association between obesity and early-onset diverticulitis, emphasizing the need for further studies to understand the underlying mechanisms. Improved patient education and adherence to treatment protocols are crucial in managing this condition and preventing complications

